# Targeted deep sequencing of circulating tumor DNA in metastatic pancreatic cancer

**DOI:** 10.18632/oncotarget.23330

**Published:** 2017-12-16

**Authors:** Andreas W. Berger, Daniel Schwerdel, Thomas J. Ettrich, Alexander Hann, Stefan A. Schmidt, Alexander Kleger, Ralf Marienfeld, Thomas Seufferlein

**Affiliations:** ^1^ Department of Internal Medicine I, Ulm University, 89081 Ulm, Germany; ^2^ Department of Diagnostic and Interventional Radiology, Ulm University, 89081 Ulm, Germany; ^3^ Institute of Pathology, Ulm University, 89070 Ulm, Germany

**Keywords:** circulating tumor DNA, liquid biopsy, pancreatic cancer, tumor evolution, tumor heterogeneity

## Abstract

**Purpose:**

Precision medicine in pancreatic ductal adenocarcinoma (PDAC) could be substantially supported by tools that allow to establish and monitor the molecular setup of the tumor. In particular, noninvasive approaches are desirable, but not validated. Characterization of circulating tumor DNA (ctDNA) may help to achieve this goal.

**Experimental Design:**

Blood samples from patients with metastatic PDAC prior to and during palliative treatment were collected. ctDNA and corresponding tumor tissue were analyzed by targeted next generation sequencing and droplet digital PCR for the 7 most frequently mutated genes in PDAC (TP53, SMAD4, CDKN2A, KRAS, APC, ATM, and FBXW7). Findings were correlated with clinical and imaging data.

**Results:**

A total of 20 patients (therapy naïve *n* = 11; pretreated *n* = 9) were included. All therapy naïve patients (*n* = 11/11) presented with detectable ctDNA at baseline. In pretreated patients, 3/7 (prior to 2nd line treatment) and 2/2 (prior to 3rd line chemotherapy) had detectable ctDNA. The combined mutational allele frequency (CMAF) of KRAS and TP53 was chosen to reflect the amount of ctDNA. The median CMAF level significantly decreased during treatment (*P* = 0.0027) and increased at progression (*P* = 0.0104). CA19-9 analyses did not show significant differences. In treatment naïve patients, the CMAF levels during therapy significantly correlated with progression-free survival (Spearman, *r* = −0.8609, *P* = 0.0013).

**Conclusions:**

Monitoring of ctDNA and its changes during treatment may enable to adapt therapeutic strategies to the specific molecular changes present at a certain time during treatment of mPDAC.

## INTRODUCTION

Pancreatic ductal adenocarcinoma (PDAC) is the most frequent malignant tumor of the pancreas [1]. Diagnosis is based on imaging and tissue analysis of the primary tumor or metastases [2]. CA19-9, the only validated tumor marker for PDAC, has a limited sensitivity (79%) and specificity (82%) [3, 4]. Due to many efforts in recent years we have now a thorough understanding of the molecular setup of PDAC and the most frequently mutated genes including *KRAS*, *TP53*, *SMAD4*, and *CDKN2A* [5–7]. In patients with metastatic PDAC (mPDAC), 1 year survival has slightly improved because of the wider use of systemic chemotherapy, but the majority of patients die within a year of diagnosis [8–10]. There is increasing evidence that systemic treatment promotes a Darwinian type of evolution of a given tumor [11, 12]. To monitor this tumor evolution, we need simple tools that allow to repetitively inform on the current molecular setup of a given tumor and could be used to guide treatment thereby improving patients’ prognosis. One promising tool for this purpose is circulating tumor DNA (ctDNA), isolated from patients´ blood. ctDNA is released into the bloodstream by mechanisms including apoptosis, necrosis and active secretion [13]. Already in patients with benign cystic pancreatic tumors we recently were able to show, that ctDNA is tumor specific [14]. Moreover, in malignancy ctDNA analysis harbors prognostic information [15, 16]. The presence of ctDNA in the plasma of patients undergoing PDAC resection indicates poor prognosis [17]. The first studies on ctDNA in PDAC focused on *KRAS* mutations that are present in the majority of PDACs [18–20]. But for treatment associated tumor evolution more genomic alterations are likely to play a role. Currently, there are only limited data available from ctDNA analyses over and above *KRAS* profiling [17, 21].

Here, we employed targeted next generation sequencing of ctDNA, combined with droplet digital PCR to examine ctDNA as (i) a tool for noninvasive diagnosis and (ii) to inform on therapy induced tumor evolution in mPDAC during different lines of systemic treatment.

## RESULTS

### Assessment of PDAC mutational profile by tissue and ctDNA analysis

**Figure 1:**
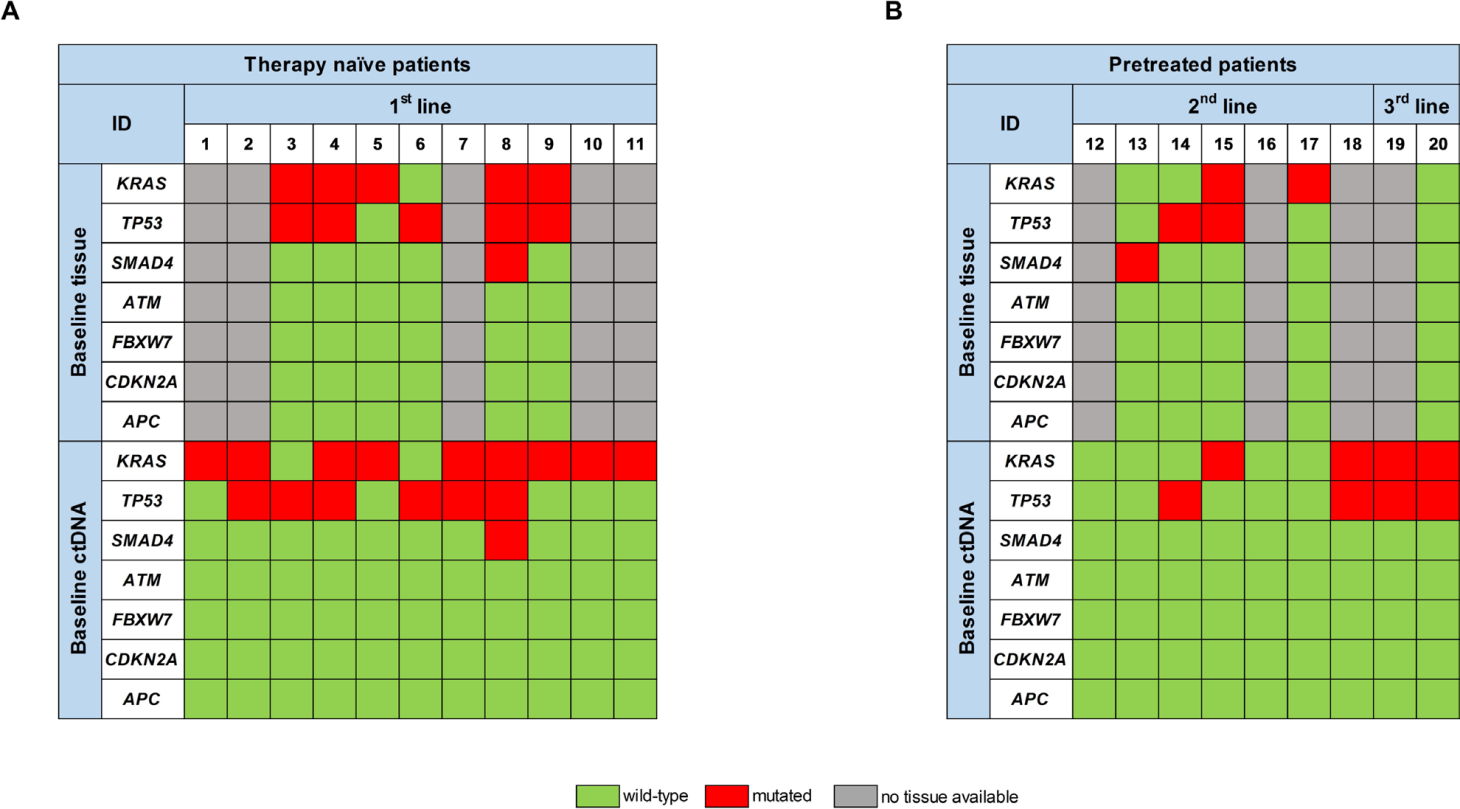
Comparison of the respective mutational spectrum detected by targeted sequencing of both archived FFPE tumor tissue material, taken at time-point of first diagnosis, and ctDNA, isolated from blood plasma at baseline, prior to initiation of the respective therapy line, for (**A**) therapy naïve patients and (**B**) pretreated patients.

At first, we analyzed ctDNA at baseline prior to initiation of the respective line of treatment. Mutations in either of the genes examined were detectable in 16/20 patients (80%). 13 patients (65%) exhibited *KRAS* mutations, 10 patients (50%) mutations in *TP53* and 1 patient (5%) a mutation in *SMAD4* (Figure 1A and 1B).

In therapy naïve patients we detected *KRAS* mutations in 9/11 patients (82%), *TP53* mutations in 6/11 patients (54%) and a *SMAD4* mutation in 1/11 patients (9%). In 6 of these patients we also analyzed tumor tissue for comparison. 5/6 tumors exhibited *KRAS* mutations (83%), 5/6 tumors *TP53* mutations and one tumor a *SMAD4* mutation. 9/11 mutations that were present in the tumor, including double mutations, could also be detected in ctDNA prior to initiation of 1st line treatment. One *KRAS* and one *TP53* mutation were only detectable in tumor tissue, resulting in an 82% tissue-blood concordance (Figure 1A).

We also analyzed ctDNA from patients in further lines of treatment (pretreated patients), where tissue from the primary tumor or metastases prior to 1st line treatment is likely to be less informative. Blood from 7 patients undergoing 2nd line and 2 patients undergoing 3rd line treatment was analyzed (Figure 1B). Prior to 2nd line treatment, we detected mutations in ctDNA of 3/7 patients (43%). From 4 of these patients FFPE material from tissue biopsy prior to 1st line treatment was available: There was at least one gene mutation detectable in the tumor tissue of all of these 4 patients. 3 of those (60%) were not detectable in ctDNA obtained prior to start of the 2nd line treatment. ctDNA analysis prior to the start of the 3rd line treatment revealed a *KRAS* and a *TP53* mutation in both patients, whereas tumor tissue from the initial diagnosis was wild type for all genes analyzed (Figure 1B).

### Effect of treatment on ctDNA allele frequencies and CA19-9 levels

**Figure 2:**
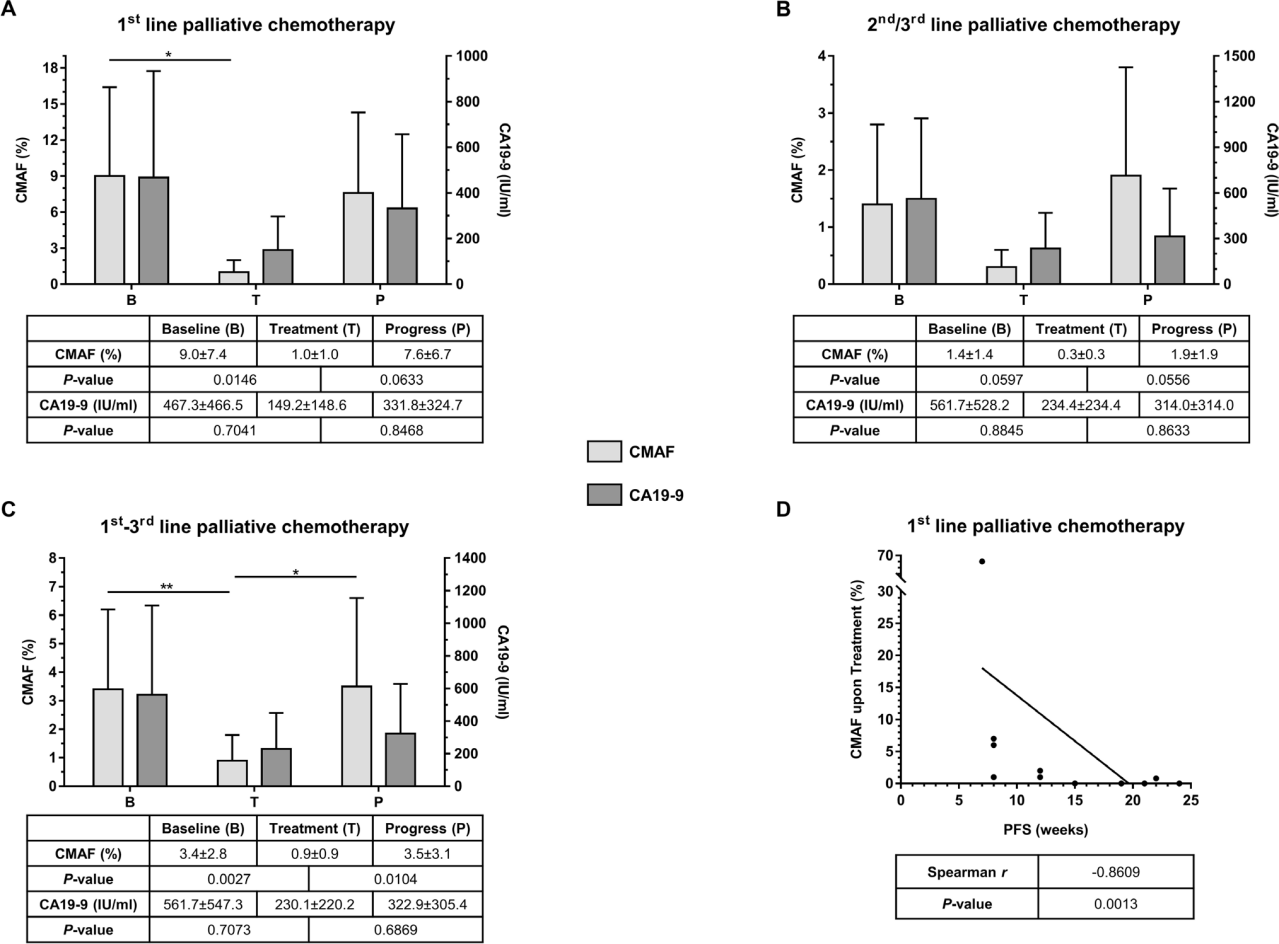
(**A**) Comparison of median CMAF and CA19-9 values at predefined time-points (B = Baseline, T = Treatment, and *P* = Progress) for patients under 1^st^ line palliative chemotherapy (*n* = 11). (**B**) Comparison of median CMAF and CA19-9 values at predefined time-points (B = Baseline, T = Treatment, and P = Progress) for patients under 2^nd^ and 3^rd^ line palliative chemotherapy (*n* = 9). (**C**) Comparison of median CMAF and CA19-9 values at predefined time-points (B = Baseline, T = Treatment, and P = Progress) for all patients of the entire cohort (*n* = 20). (**D**) Correlation of CMAF upon treatment with progression-free survival (PFS); CMAF = combined mutational allele frequency of *KRAS* and *TP53*, values are %, given as median ± median absolute deviation

Since all patients with detectable ctDNA at baseline had either a *KRAS* or a *TP53* mutation and 96% of the mutations in ctDNA constituted either a *KRAS* or a *TP53* mutation, the mutated allele frequencies (MAF) of these two genes were selected to form a combined MAF score (CMAF) that reflects the amount of ctDNA. In therapy naïve patients the median CMAF at baseline was 9.0% ± 7.4% and decreased significantly to 1.0% ± 1.0% upon treatment (Figure 2A, *P* = 0.0146). At disease progression, the median CMAF increased again to 7.6% ± 6.7% (*P* = 0.0633). The CA19-9 median baseline level was 467.25 IU/ml ± 466.45 IU/ml and decreased to 149.20 IU/ml ± 148.55 IU/ml (*P* = 0.7041) upon treatment. CA19-9 also increased at disease progression to 331.80 IU/ml ± 324.70 IU/ml. However, these differences in CA19-9 were not significant (*P* = 0.8468, Figure 2A).

In the cohort of patients that had already received chemotherapy the median CMAF at baseline, prior to initiation of the respective line of therapy was 1.4% ± 1.4%. This level decreased upon treatment to 0.3% ± 0.3% (Figure 2B, *P* = 0.0597). At disease progression, the median CMAF increased again to 1.9% ± 1.9% (*P* = 0.0556). In this situation, the median baseline level of CA19-9 was 561.7 IU/ml ± 528.2 IU/ml, and decreased to 234.4 IU/ml ± 234.4 IU/ml upon treatment (*P* = 0.8845). At disease progression CA19-9 raised again to 314.0 IU/ml ± 314.0 IU/ml (*P* = 0.8633, Figure 2B).

In summary, across all lines of treatment, CMAF dynamics, but not absolute values clearly reflected tumor evolution during palliative chemotherapy. Among the entire cohort, the median CMAF level significantly decreased (*P* = 0.0027) during treatment and, conversely, significantly increased again at disease progression (*P* = 0.0104; Figure 2C). CA19-9 analyses showed a similar trend but did not reach statistical significance (Figure 2C). In treatment naïve patients, the decline in the CMAF levels during treatment was predictive: A decline in CMAF in the ctDNA during therapy significantly correlated with progression-free survival of the patients (Spearman, *r* = −0.8609, *P* = 0.0013; Figure 2D). Of note, no differences in CMAF value and its change or PFS between the therapeutic regimen in 1st line treatment could be observed (data not shown).

### Assessing PDAC evolution by sequential ctDNA analysis

**Figure 3:**
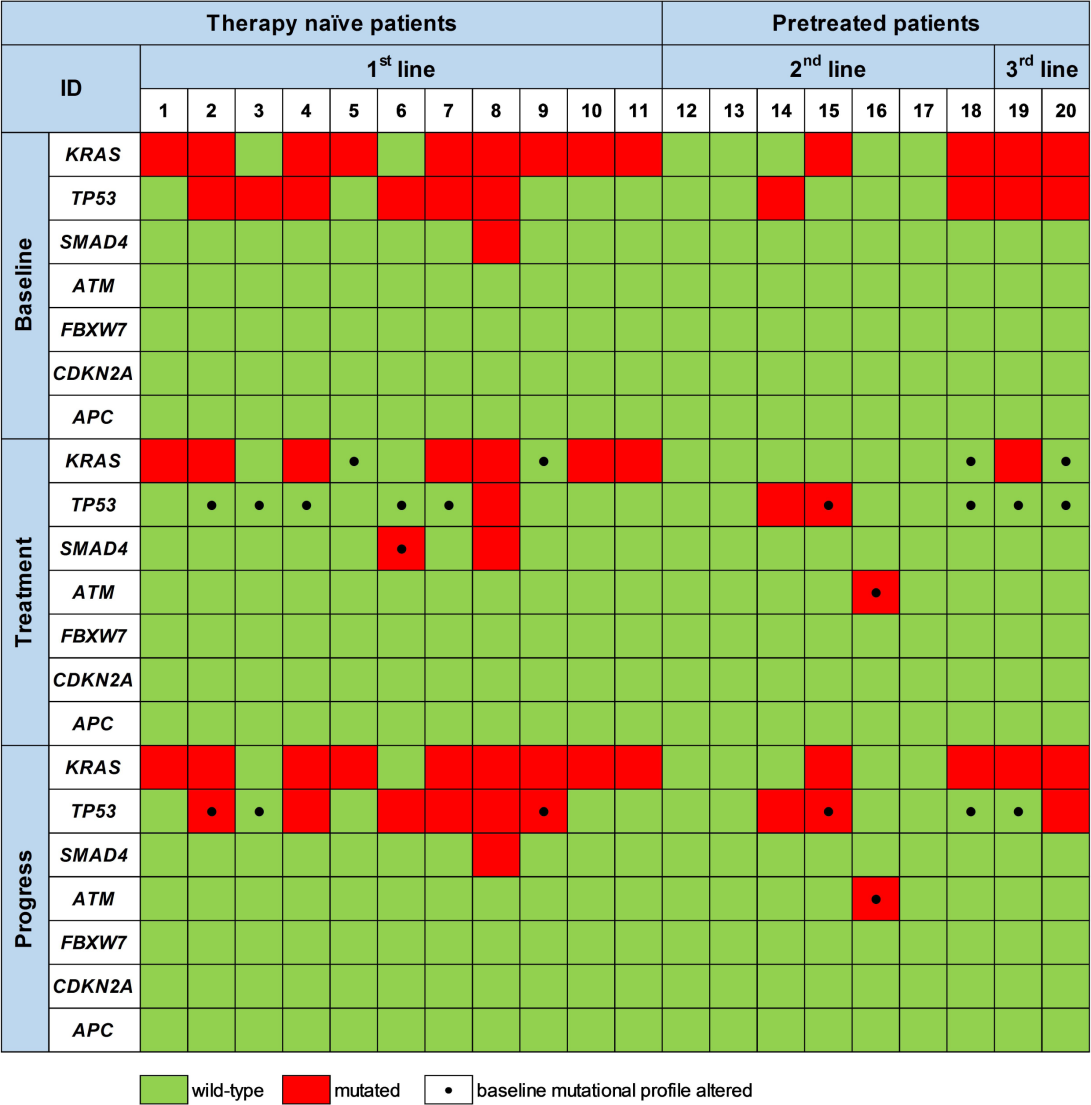
Mutations detected by targeted sequencing of ctDNA prior to therapy initiation (Baseline), upon treatment (Treatment) and at disease progression (Progress) Black dots at the respective time-point mark mutations that differ from the initial mutational profile generated at baseline prior to initiation of the respective therapy line.

**Figure 4:**
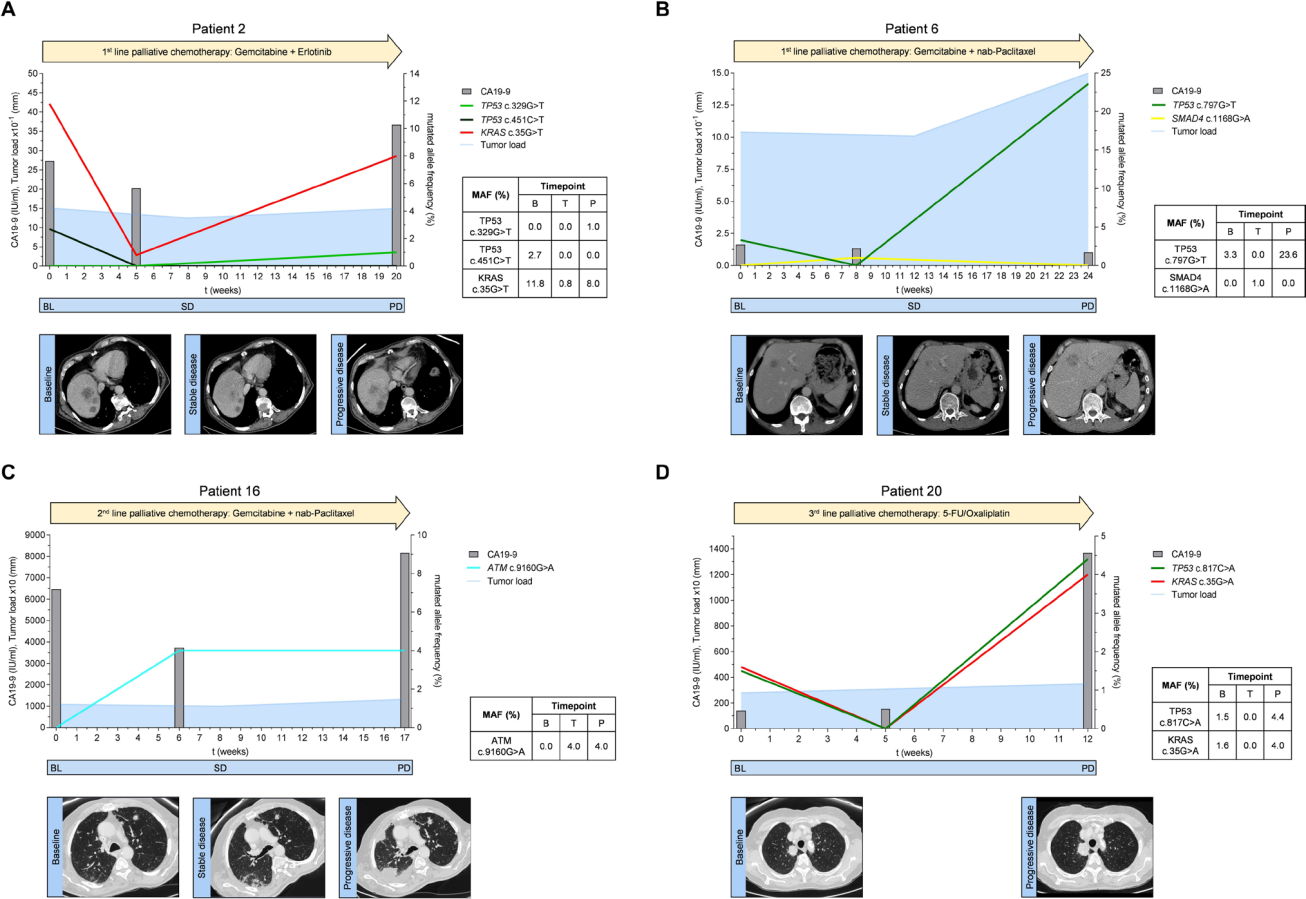
(**A–D**) Mutated allele frequencies, tumor marker CA19-9 levels and tumor load according to RECIST 1.1 for exemplary patients spanning the respective therapy line from baseline before therapy initiation to disease progression; BL = baseline, SD = stable disease, PD = progressive disease.

By sequential ctDNA analyses during 1st line palliative chemotherapy we detected a change in the mutational landscape in 7/11 patients (64%) compared to baseline. A similar pattern was observed during 2nd and 3rd line treatment, where 3/7 (43%) and 2/2 (100%) of the patients exhibited a change in the mutational pattern of ctDNA compared to the baseline ctDNA status (Figure 3). This is shown in more detail for four patients in Figure 4A–4D.

Patient 2 received 1st line palliative chemotherapy with gemcitabine and erlotinib (Figure 4A). Prior to treatment initiation, ctDNA analysis revealed a *TP53* mutation (c.451C>T) and a *KRAS mutation* (c.35G>T). The mutational frequency of both *TP53* and *KRAS* mutations dropped remarkably during treatment. This suggested a change in the clonal composition of the tumor that resulted in disease control (stable disease) as confirmed by a CT 8 weeks after therapy initiation. Cytotoxic chemotherapy in this patient obviously eliminated the *TP53* mutated clone(s) (c.451C>T) which did not recur at disease progression 5 months after start of chemotherapy. However, at disease progression a new *TP53* mutation (c.329G>T) was detectable in ctDNA. Also, the allele frequency of the *KRAS* mutation was markedly increased at that stage. CA19-9 levels showed a moderate drop at week 5 after initiation of treatment and increased again at progression.

Patient 6 received 1st line palliative chemotherapy with gemcitabine and nab-paclitaxel (Figure 4B). At baseline, ctDNA analysis showed a *TP53* mutation (c.797G>A) which had also been detected in tumor tissue. 8 weeks after the start of treatment, the *TP53* mutation was no longer detectable by ctDNA analysis and a CT scan at week 12 showed stable disease. Interestingly, a *SMAD4* mutation (c.1168G>A) became detectable during treatment. At disease progression, the initial *TP53* mutation was again detectable whereas the *SMAD4* mutation disappeared. The CA19-9 value was not elevated at baseline and did also not change during the course of therapy (<32 IU/ml).

Patient 16 received gemcitabine and nab-paclitaxel as 2nd line treatment (Figure 4C). Prior to start of 2nd line treatment no mutations in the analyzed genes were detectable in ctDNA. After six weeks of treatment we observed a mutation in the *ATM* gene (c.9160G>A). The CA19-9 level was lower compared to baseline. At week 8 CT-scan suggested stable disease. However, a subsequent CT scan 9 weeks later stated disease progression and CA19-9 was elevated. The *ATM* mutation (c.9160G>A), but no other mutation was again detectable in ctDNA.

Patient 20 received 5-FU/oxaliplatin as a 3rd line palliative chemotherapy (Figure 4D). Tumor tissue from the initial diagnosis (primary tumor after resection) did not show any mutation in all analyzed genes. Conversely, the initial ctDNA analysis prior 3rd line treatment exhibited two mutations, one in *TP53* (c.817C>A) and one in *KRAS* (c.35G>A) suggesting clonal selection during the previous lines of treatment. ctDNA analysis did not show these mutations at the first examination during treatment suggesting that cytotoxic chemotherapy in this patient temporarily suppressed the mutated *TP53* and *KRAS* alleles. However, this effect was only transient and both mutations were again detectable at high levels 12 weeks after start of 3rd line chemotherapy when the disease was progressing. Concurrently, CA19-9 levels also increased at this time.

## DISCUSSION

In 1983, Shapiro *et al*. were the first to report the presence of circulating cell-free DNA in the blood of patients with PDAC [22]. More than 20 years later other groups isolated and analyzed ctDNA (in particular *KRAS* genotyping) from patients with PDAC both in the metastatic [21] and the curative setting [23]. Here we examined the potential of targeted ctDNA genotyping beyond *KRAS* profiling in patients with metastatic PDAC in distinct clinical situations (therapy naïve and pretreated patients).

Among all analyzed genes in this study, we detected tumor-specific mutations in ctDNA in all treatment-naïve patients with 82% blood-tissue concordance. These data from a small cohort stress the power of ctDNA as a noninvasive tool for tumor diagnosis. Recently it was reported that only 48% of patients with advanced PDAC had detectable ctDNA. In this report the authors pooled the data of locally advanced and metastatic PDAC which may be responsible for the low percentage of ctDNA positivity [17]. In our study, only metastatic PDAC was analyzed. In the 2nd line setting, we detected fewer mutations in ctDNA than in the primary tumors. This may be due to the small sample size, but also due to a different clonal composition of the tumor in 2nd line compared to the primary tumor. Treatment could have largely eradicated these clones during 1st line treatment or the respective clones were not any more affected by the respective treatment and thereby there was no ctDNA released from apoptotic or necrotic cells, disabling analysis of ctDNA. We did not perform repeated tumor biopsies to further investigate this issue since they are cumbersome for the patient and may also yield only limited results due to intratumoral heterogeneity and consequently sampling errors as has been described for tumors [24]. Nevertheless, in the more advanced tumors (3rd line situation) ctDNA revealed mutations that were not detected by the analysis of the primary tumor. Thus, analysis of ctDNA gives an overview on the mutational state of the tumor at a certain time during treatment. Nevertheless, the sample capacity is low, thus the result of statistical analysis should be interpreted with caution and needs further validation.

Since ctDNA is easy to obtain, it may allow for monitor clonal evolution during chemotherapy. But data for PDAC are sparse [17, 19, 21, 25]. Single gene analysis for capturing of tumor evolution in mPDAC may be insufficient. Interestingly, we predominantly detected mutations in *KRAS* and *TP53* in ctDNA but rarely mutations in other genes such as *SMAD4* or *ATM*. This may also be due to the small sample size analyzed but also reflects the marked heterogeneity of PDAC. Previous studies reported on panels of 54 [21] or 22 [17] genes, but at least *KRAS* and *TP53* also remained the most frequently mutated genes. Thus, we still need to define the optimal approach and/or gene set to define the molecular setup of a given tumor by ctDNA analysis. For assessing prognosis the number of mutated genes in ctDNA seems to have no impact as recently reported [17].

Given the frequent mutation of *KRAS* and *TP53* we used these mutations to build a score, CMAF. This score performed better in assessing tumor evolution than CA19-9. Examination of *KRAS* and *TP53* mutations in ctDNA over the course of treatment revealed, that upon disease stabilization during 1st line treatment, *TP53* mutations were frequently not any more detectable. This could be due to suppression or even eradication of *TP53* mutated clones by treatment. Interestingly, in the majority of cases a *TP53* mutation reappeared upon disease progress in line with the well-known function of *TP53* in tumor propagation [26]. The observed *TP53* mutations in our case reports (Figure 4A and 4B) were recently described to be pathogenic in PDAC [27, 28]. *TP53* plays also a role over the course of several lines of treatment. E.g. in patient 20 (Figure 4D), two novel mutations in *KRAS* and *TP53* were detectable in ctDNA prior to 3rd line treatment suggesting that in some cases the initial *TP53* clone was indeed eradicated by treatment but there was a selection of other low frequency alleles during therapy.

As mentioned, most of the mutations detectable either in tissue or in ctDNA were in *KRAS* and *TP53* and mutations in other genes such as *SMAD4* were only rarely detectable. Interestingly, these mutations may not always have an impact on disease progression since in one case a *SMAD4* mutation occurred during treatment but was not detectable any more upon disease progress. Thus, tumor evolution is a dynamic system that has indeed traits of a Darwinian evolution with mutations that do not provide an advantage for the tumor disappearing whereas others that provide an advantage reappear and get selected.

Analysis of ctDNA in PDAC may in some cases provide an opportunity to guide treatment over time: In one patient (patient 16, Figure 4C), we detected a mutation in the *ATM* gene at the time of progression during 2nd line treatment with gemcitabine and nab-paclitaxel. This distinct mutation may have conferred resistance to the present treatment and is so far not described yet. Nevertheless, this may also make the tumor vulnerable to PARP inhibition [29], in particular since there was no *TP53* mutation detectable either by tissue or by ctDNA analysis.

In conclusion, these data open the avenue for a molecular staging of PDAC using ctDNA both in therapy naïve and pretreated patients. ctDNA analysis can complement and extend tissue analysis and radiological assessment. The use of informed panels for targeted resequencing is likely to be insufficient for a comprehensive assessment of treatment induced PDAC evolution as a basis for a rational choice of a targeted treatment strategy. This is most likely due to the fact that driver mutations in PDAC are rather heterogeneous and occur at a low frequency in the whole set of tumors. Therefore, a more extensive analysis of ctDNA by whole exome sequencing and the incorporation of additional data from epigenetics and/or the metabolome maybe crucial to achieve this goal. Further prospective analyses are warranted to substantiate these hypotheses.

## MATERIALS AND METHODS

### Institutional review board

Prior to start of the study a positive vote from the institutional review board of Ulm University was obtained (Ulm University, Approval numbers: 317/12, 230/14, 128/15). Participation in the study was voluntary. All patients signed a written informed consent prior to inclusion.

### Patient characteristics and study design

Twenty patients with histologically confirmed metastatic PDAC (UICC stage IV) were enrolled in this study. Patient characteristics are shown in Supplementary Data File 1. All patients received palliative chemotherapy: 11 patients were analyzed during 1st line, 7 patients during 2nd line and 2 patients during 3rd line treatment. Blood samples for ctDNA analyses were taken prospectively at predefined time points (“Baseline”: prior to treatment initiation; “Treatment”: 4.4 ± 0.4 weeks after treatment initiation; “Progress”: at radiologically confirmed disease progression). Archived FFPE tumor material from initial diagnosis was used for comparison. All ctDNA and tumor tissue DNA samples were analyzed by targeted next generation sequencing (NGS). The mutational status was validated by droplet digital PCR (ddPCR) when the NGS analysis of *KRAS* and *TP53* was discordant between tumor tissue and ctDNA. Details of the molecular characteristics of the tumor tissue and ctDNA are provided in Supplementary Data File 2. CT-scans were done at baseline and at a mean of 9.6 ± 0.7 weeks during treatment (all according to RECIST 1.1). CA199 measurements were performed in parallel and at the same time points as ctDNA analyses (Roche Diagnostics Germany, Mannheim, Germany, normal value: <32 IU/ml).

### Plasma collection

7.5 ml of whole venous blood were collected in EDTA tubes (Sarstedt, Nümbrecht, Germany) by peripheral blood draw, kept at 4°C until separation (within one hour after collection). Whole blood was centrifuged for 10 minutes (820 × g at 4°C), plasma fraction was transferred to cold 2ml tubes (Eppendorf RNA/DNA LoBind micro-centrifuge tubes, Eppendorf, Hamburg, Germany) and subsequently centrifuged again for 10 min (20.000 × g at 4°C). Pure plasma was recovered in fresh 2 ml tubes for immediate storage at −80°C until ctDNA extraction.

### Extraction of ctDNA

ctDNA was extracted from plasma using the QIAamp Circulating Nucleic Acid Kit (QIAGEN, Hilden, Germany) according to the manufacturer's instructions. For each patient, we used 2 ml of plasma for ctDNA extraction and recovered ctDNA in 50 μl of elution buffer. ctDNA was stored at −20°C until further use.

### Isolation of tumor DNA from FFPE tissue

For isolation of genomic DNA from the FFPE tissue samples, 5 μm tissue slices were transferred to glass slides. To estimate the area containing the tumor, HE stained FFPE tissue slices (2 μm) were validated by an expert pathologist. The tumor-harboring areas of the FFPE tissue was subjected to a DNA extraction procedure using the QIAamp DNA FFPE tissue kit (QIAGEN, Hilden, Germany) according to manufacturer's instruction. DNA purity and concentration was determined fluorometrically (Qubit 2.0; Invitrogen, Carlsbad, CA, USA).

### Next generation sequencing

For molecular characterization of both tumor tissue and ctDNA, we employed a targeted re-sequencing methodology using the GeneRead V2 chemistry (QIAGEN, Hilden, Germany) and a custom-made re-sequencing panel including primers for all exons of *TP53, SMAD4, CDKN2A, KRAS, APC, ATM*, and *FBXW7* (primer sequences and locations of target areas are available upon request). The mentioned genes were selected according to previously published data, covering the most frequently mutated genes in PDAC [5, 6]. Target enrichment, amplicon processing, and library generation were performed according to the manufacturer's instructions. For target enrichment, we included 1ng (ctDNA) to 40 ng (DNA from FFPE tumor tissue). Successful target enrichment and library generation was checked using the High Sensitivity DNA kit on a bioanalyzer device (Agilent, Santa Clara, CA, USA). Libraries were diluted to 10 pM solutions and the sequencing was performed on a MiSeq platform (Illumina, San Diego, CA, USA) using the V2 chemistry. Mean read depth on target region was 2000–8000 fold and 99% of bases were covered at 96–100% on average. The resulting fastq files were subjected to further analysis using the GeneRead web based analysis tool (http://ngsdataanalysis.sabiosciences.com/NGS2/), the Biomedical Workbench software package (QIAGEN, Hilden, Germany), and the Variant Studio software (Illumina, San Diego, CA, USA). For the analysis of the fastq files and thus for the calling of the mutations we also used the CLC Biomedical Workbench. To be more precise, we used a Somatic Cancer (TAS) workflow termed Identify Variants (TAS). In this workflow variants are detected using the Low Frequency Variant Detection tool, which relies on statistical models to minimize calling of false positives. These two statistical models are a statistical model for the analyzed sample and a model for the sequencing errors described in more detailed in the CLC Biomedical Workbench manual (http://resources.qiagenbioinformatics.com/manuals/biomedicalgenomicsworkbench/current/index.php?manual=_Low_Frequency_Variant_caller_Models_methods.html). Moreover, the level of detection (LOD) of 1% was verified by spike in experiments using the Multiplex I cfDNA Reference Standard Set by (Horizon^®^).

### Droplet digital PCR (ddPCR) analyses

Isolated ctDNA was amplified using ddPCR™ Supermix for Probes (Bio-Rad^®^, Hercules, CA, USA) and the respective PrimePCR™ ddPCR™ Mutation Assay (Bio-Rad^®^, Hercules, CA, USA). 8μl of eluate were used in each reaction and mixed with 2μl of primers/probes and 10μl of Supermix. The reaction mix was then vortexed and immediately transferred into a DG8™ Cartridge together with 70μl of Droplet Generation Oil for Probes for droplet generation in a QX200™ Droplet Generator (all: Bio-Rad^®^, Hercules, CA, USA). Droplets were carefully transferred into a 96-well plate, which was sealed with PX1™ PCR Plate Sealer for subsequent amplification in a T100™ Thermal Cycler according to the manufacturer's instructions (all: Bio-Rad^®^, Hercules, CA, USA). Droplets were analyzed in QX200™ Droplet Reader (Bio-Rad^®^, Hercules, CA, USA) for fluorescent measurement of FAM and HEX probes. Gating was based on DNA standard (50% WT, 50% Mutant) by Horizon^®^ and H_2_0 as no-template-control. Thresholding was done based on positive and negative controls for each assay. False-positive-rates (FPR) were determined for each assay individually using wild-type reference DNA (Horizon^®^) in appropriate concentrations. Samples were called positive based on Poisson distribution when reaching 99% confidence level for being positive. Digital PCR data was analyzed by QuantaSoft analysis software (version 1.7.4) according to the manufacturer's instructions (Bio-Rad^®^, Hercules, CA, USA).

### Statistical analyses

Statistical analyses are based on a descriptive, hypothesis-generating approach. Results for continuous variables are presented as median ± median absolute deviation (MAD) or mean ± standard error of the mean (SEM) unless stated otherwise. Treatment groups were compared with the Mann-Whitney *U*-test. Comparison of categorical variables was generated by the Pearson χ^2^ test. Correlation analyses were performed by Pearson or Spearman correlation analysis, *P* values < 0.05 were considered significant. All statistical analyses were performed using GraphPad Prism version 7 (GraphPad Software, La Jolla, CA, USA).

## SUPPLEMENTARY MATERIALS TABLES



## Abbreviations

μl: Microliter
μm: Micrometer
APC: Adenomatous polyposis coli
ATM: ATM serine/threonine kinase
CA 19-9: Carbohydrate-Antigen 19-9
CDKN2A: Cyclin-Dependent Kinase Inhibitor 2A
CMAF: Combined Mutational Allele Frequency
CT: Computed Tomography
ctDNA: circulating tumor DNA
ddPCR: droplet digital Polymerase Chain Reaction
EDTA: Ethylenediaminetetraacetic Acid
FBXW7: F-box and WD repeat domain containing 7
FFPE: Formalin-Fixed, Paraffin-Embedded
FPR: False Positive Rate
IU/ml: International Unit/milliliter
KRAS: V-Ki-ras2 Kirsten rat sarcoma
MAD: Median Absolute Deviation
MAF: Mutational Allele Frequency
Min: Minute
mPDAC: metastatic Pancreatic Ductal Adenocarcinoma
ng: nanogram
NGS: Next Generation Sequencing
PCR: Polymerase Chain Reaction
PDAC: Pancreatic Ductal Adenocarcinoma
pM: pico Molar
RECIST: Response Evaluation Criteria in Solid Tumors
SEM: Standard Error of the Mean
SMAD4: SMAD family member 4
TP53: Tumor Protein 53
UICC: Union Internationale Contre le Cancer
WT: Wildtype
