# Supplementary material for: Targeted deep sequencing of circulating tumor DNA in metastatic pancreatic cancer
Source: Oncotarget. 2017 Dec 16;9(2):2076–85. doi: 10.18632/oncotarget.23330 (PMC5788622; doi:10.18632/oncotarget.23330)
Supplement: Supplementary file 1 [file oncotarget-09-2076-s001.pdf]

Targeted deep sequencing of circulating tumor DNA in metastatic pancreatic cancer

SUPPLEMENTARY MATERIALS

Supplementary Data File 1: Patient characteristics of the entire study cohort

ID	Sex	Age (years)	Stage	Metastases	Therapy line	Therapy regimen	PFS (weeks)
1	m	64	UICC IV	HEP	1st line	Gemcitabine/Erlotinib	12
2	m	75	UICC IV	HEP, LYM, PER	1st line	Gemcitabine/Erlotinib	22
3	w	67	UICC IV	HEP	1st line	Gemcitabine/Erlotinib	21
4	m	72	UICC IV	HEP	1st line	Gemcitabine/nab-Paclitaxel	8
5	m	72	UICC IV	HEP, PER, OSS	1st line	Gemcitabine/nab-Paclitaxel	19
6	m	60	UICC IV	PER	1st line	Gemcitabine/nab-Paclitaxel	24
7	m	62	UICC IV	HEP	1st line	FOLFIRINOX	12
8	w	79	UICC IV	HEP	1st line	Gemcitabine/Erlotinib	7
9	m	50	UICC IV	PUL	1st line	Gemcitabine/Erlotinib	15
10	m	62	UICC IV	HEP, PER	1st line	FOLFIRINOX	8
11	w	73	UICC IV	HEP, PUL	1st line	Gemcitabine/nab-Paclitaxel	8
12	w	61	UICC IV	HEP, PUL	2nd line	FOLFIRINOX	10
13	m	78	UICC IV	PUL	2nd line	FOLFIRI	8
14	m	75	UICC IV	PUL	2nd line	5FU/Oxaliplatin	24
15	m	85	UICC IV	HEP	2nd line	5FU/Oxaliplatin	49
16	w	44	UICC IV	HEP	2nd line	Gemcitabine/nab-Paclitaxel	19
17	m	59	UICC IV	HEP	2nd line	Gemcitabine/nab-Paclitaxel	20
18	m	73	UICC IV	HEP	2nd line	Docetaxel/Oxaliplatin	31
19	m	58	UICC IV	HEP, PER	3rd line	Docetaxel/Oxaliplatin	11
20	w	76	UICC IV	PER	3rd line	5FU/Oxaliplatin	25

UICC = union international contre le cancer, 5FU = 5-Fluorouracil, PFS = progression-free survival.

Supplementary Data File 2: Detailed molecular data of the entire study cohort

ID	Variant	Tissue mutated	ctDNA mutated allele frequency (%)		
			Baseline	Treatment	Progress
1	*KRAS* G12V (c.35G>T)	n.a.	0.8	2.0	0.9
2	*KRAS* G12V (c.35G>T)	n.a.	11.8	0.8	8.0
	TP53 P151S (c.451C>T)	n.a.	2.7	0	0
	TP53 R110L (c.329G>T)	n.a.	0	0	1.0
3	*KRAS* G12V (c.35G>T)	yes	0	0	0
	TP53 R175H (c.524G>A)	yes	2.0	0	0
4	*KRAS* G12D (c.35G>A)	yes	9.0	1.0	1.9
	TP53 R282W (c.844C>T)	yes	11.0	0	3.0
5	*KRAS* G12D (c.35G>A)	yes	9.0	0	1.0
6	*TP53* G266V (c.797G>T)	no	3.3	0	23.6
	SMAD4 Q390K (c.1168G>A)	no	0	1.0	0
7	*KRAS* G12D (c.35G>T)	n.a.	3.5	1.0	10.0
	TP53 N263fs (c.788delA)	n.a.	2.8	0	8.5
8	*KRAS* G12V (c.35G>T)	yes	64.0	41.0	69.6
	TP53 R273H (c.818G>A)	yes	42.0	27.0	52.0
	SMAD4 R361H (c.1082G>A)	yes	42.0	22.0	51.0
9	*KRAS* G12D (c.35G>A)	yes	1.3	0	5.6
	TP53 R248Q (c.743G>A)	yes	0	0	2.0
10	*KRAS* G12D (c.35G>A)	n.a.	47.0	7.0	8.0
11	*KRAS* G12D (c.35G>A)	n.a.	9.0	6.0	1.1
12	-	n.a.	-	-	-
13	*SMAD4* R361H (c.1082G>A)	yes	0	0	0
14	*TP53* P72R (c.215C>G)	yes	1.0	1.0	1.0
15	*KRAS* A146V (c.437C>T)	yes	0.39	0	1.1
	TP53 R175H (c.524G>A)	yes	0	1.0	1.0
16	*ATM* A3054T (c.9160G>A)	n.a.	0	4.0	4.0
17	*KRAS* G12R (c.34G>C)	yes	0	0	0
18	*KRAS* G12V (c.35G>T)	n.a.	1.4	0	0.8
	TP53 H214Y (c.640C>T)	n.a.	1.0	0	0
19	*KRAS* G12V (c.35G>T)	n.a.	2.4	0.3	1.9
	TP53 F19S (c.56T>C)	n.a.	1.1	0	0
20	*KRAS* G12D (c.35G>A)	no	1.6	0	4.0
	TP53 R273S (c.817C>A)	no	1.5	0	4.4

n.a.= not available.
